# Supplementary material for: Translational coupling via termination-reinitiation in archaea and bacteria
Source: Nat Commun. 2019 Sep 5;10:4006. doi: 10.1038/s41467-019-11999-9 (PMC6728339; doi:10.1038/s41467-019-11999-9)
Supplement: Supplementary file 1 — Supplementary Information [file 41467_2019_11999_MOESM1_ESM.pdf]

Supplementary Information:

Translational coupling via termination-reinitiation in archaea and bacteria

Huber et al. (2019)

Nature Communications

Supplementary Table 1. List of overlapping gene pairs with designations and names of u pstream and downstream genes and sequences around the overlap. The SD motifs are shown i n red, the start codons in blue, and the stop codons in bold.

	Gene Designation	Gene ubstream	Sequence (overlap)	Gene Designation	Gene downstream	Plasmid
H. volcanii	HVO_0147	*ureB*	CGGCACCGCGGAGGGGTCGAGCGAA**TGA**CGAAGGAC	HVO_0148	*ureC*	pEK2
	HVO_0357	*aa-bin. Prot.*	ACCGCTCGCGGAGGGGTCCCAA**TGA**GCCTCAGACTC	HVO_0358	*hom1*	pEK3
	HVO_2543	*rpl30*	CGAACTCCTGGAGGACATGCGA**TGA**CGTCCAAGAAG	HVO_2542	*rpl15*	pEK5
	HVO_2551	*rpl5*	GTTCGACGTGGAGGTTGAAGAA**TGA**GCGATAGCGAA	HVO_2550	*rps15*	pEK6
	HVO_1210	*flgA1*	ATTGCGCTC**TGA**GGAGATTCAAATGTTCAACAACAT	HVO_1211	*flgA2*	pEK4
	HVO_2431	*glnP*	TCGGATTGGGGAGGTGACCGCCGA**TGA**CGCTCGTC	HVO_2430	*glnQ*	pIM1
	HVO_1594	*cna*	CGCCAAACTGGAGGTGACCGCA**TGA**CGCTCGTCTC	HVO_1595	*con. hyp. prot.*	pIM2
	HVO_0685	*hyp. prot.*	GCGGCCGACGGAGGTGAG**TGA**GATGGAACTCGTCT	HVO_0686	*con. hyp. prot.*	pIM3
	HVO_2555	*rps17*	GAGATTATGGGAGGTGACGAG**TGA**TGGAACTCGTC	HVO_2554	*rpl14*	pIM4
E. coli	b2488	*hyfH*	CTGCTGGTGGCTAAGGAGCAGCTA**TGA**GTCCAGTGC	b2489	*hyfI*	pMH1
	b1746	*astD*	GATTTTTCCGATGAGGTGGTGCGA**TGA**ACGCCTGGG	b1745	*astB*	pMH2
	b0775	*bioB*	AATATTACAACGCGGCAGCATTA**TGA**GCTGGCAGGA	b0776	*bioF*	pMH3
	b1381	*ybdH*	GGAAAAGAGTGTGAGGAAAAACAA**TGA**AAATTTTAC	b1382	*ynbE*	pMH4
	b2264	*menD*	TCTGGCGCAGGTAAGCCATTTA**TGA**TCCTGCACGCG	b2263	*menH*	pMH5

Supplemtary Table 2. Native (wt) and mutated (mut) sequences of analyzed overlapping gene regions in *H. volcanii* and *E. coli*. Shine-Dalgarno regions are underlined, matching nucleotides to the consensus sequence are indicated in red, start codon of downstream gene indicated in blue and stop codon in bold.

	HVO-Nummer	Gen upstream		Sequence (overlap)	HVO-Nummer	Gen downstream
H. volcanii	HVO_0147	*ureB*	wt	CGGCACCGCGGAGGGGTCGAGCGAATGACGAAGGAC	HVO_0148	*ureC*
			mut	CGGCACCGCTAGTAGCGCGAGCGAATGACGAAGGAC		
	HVO_0357	*aa-bin. Prot.*	wt	ACCGCTCGCGGAGGGGTCCCAATGAGCCTCAGACTC	HVO_0358	*hom1*
			mut	ACCGCTCGCTAGTAGCGCCCAATGAGCCTCAGACTC		
	HVO_2543	*rpl30*	wt	CGAACTCCTGGAGGACATGCGATGACGTCCAAGAAG	HVO_2542	*rpl15*
			mut	CGAACTCCTTAGTAGCGTGCGATGACGTCCAAGAAG		
	HVO_2551	*rpl5*	wt	GTTCGACGTGGAGGTTGAAGAATGAGCGATAGCGAA	HVO_2550	*rps15*
			mut	GTTCGACGTTAGTAGCGAAGAATGAGCGATAGCGAA		
	HVO_2431	*glnP*	wt	TCGGATTGGGGAGGTGACCGCCGATGACGCTCGTCT	HVO_2430	*glnQ*
			mut	TCGGATTGGCTCCTCAGCCGCCGATGACGCTCGTCT		
	HVO_1594	*cna*	wt	CGCCAAACTGGAGGTGACCGCATGACGCTCGTCTCT	HVO_1595	*con. hyp. prot.*
			mut	CGCCAAACTCTCCTCAGCCGCATGACGCTCGTCTCT		
	HVO_0685	*hyp. prot.*	wt	GCGGCCGACGGAGGTGAG**TGA**GATGGAACTCGTCTC	HVO_0686	*con. hyp. prot.*
			mut	GCGGCCGACCTCCTCAGG**TGA**GATGGAACTCGTCTC		
E. coli	b2488	*hyfH*	wt	CTGCTGGTGGCTAAGGAGCAGCTATGAGTCCAGTGC	b2489	*hyfI*
			mut	CTGCTGGTGGCAAGCTTGCAGCTATGAGTCCAGTGC		
	b1746	*astD*	wt	GATTTTTCCGATGAGGTGGTGCGATGAACGCCTGGG	b1745	*astB*
			mut	GATTTTTCCGACACCACCTTGCGATGAACGCCTGGG		
	b0775	*bioB*	wt	AATATTACAACGCGGCAGCATTATGAGCTGGCAGGA	b0756	*bioF*
			mut	AATATTACAAATTATTGTTGTTATGAGCTGGCAGGA		
	b1381	*ybdH*	wt	GGAAAAGAGTGTGAGGAAAAACAATGAAAATTTTAC	b1382	*ynbE*
			mut	GGAAAAGAGTGTGCACCACTGCAATGAAAATTTTAC		
	b2264	*menD*	wt	TCTGGCGCAGGTAAGCCATTTATGATCCTGCACGCG	b2263	*menH*
			mut	TCTGGCGCAGGCTGCAGATTTATGATCCTGCACGCG		

Supplementary Table 3. Oligonucleotides for amplification of overlapping gene pairs.

HVO-Nummer	Gen upstream		Sequence (primer 5'-3‘ for genepair amplification)	HVO-Nummer	Gen downstream
HVO_0147	*ureB*	fw	AATTCAATTGATGACCGGCGAGTTCGTTCC	HVO_0148	*ureC*
		rev	CATGCCATGGGAACAGTTCGGTGTCG		
HVO_0357	*aa-bin. Prot.*	fw	AATTCAATTGATGAGCGCACAGGACCTCGA	HVO_0358	*hom1*
		rev	CATGCCATGGGGCGACGACCTCG		
HVO_2543	*rpl30*	fw	AATTCAATTGATGCAGGCTATCGTTCAGCT	HVO_2542	*rpl15*
		rev	CATGCCATGGACCGCCGCGGTGA		
HVO_2551	*rpl5*	fw	AATTCAATTGATGAGCGAGGCTGACTTCCA	HVO_2550	*rps15*
		rev	CATGCCATGGCTGCTTTCGACCGCAG		
HVO_1210	*flgA1*	fw	AATTCAATTGATGTTCGAAAACATCAACGA	HVO_1211	*flgA2*
		rev	CATGCCATGGGATTGCGGCGACCA		
HVO_2431	*glnP*	fw	CGAACTCTGCAGTATGGCAGACACATACTCAGGGG	HVO_2430	*glnQ*
		rev	CAGAGACGAGCGTCATCGGCGGTCACCTCCCCAATCC		
HVO_1594	*cna*	fw	GAACTCTGCAGTATGAACCCGCTCCAGCGG	HVO_1595	*con. hyp. prot.*
		rev	CAGAGACGAGCGTCATGCGGTCACCTCCAGTTTGGCG		
HVO_0685	*hyp. prot.*	fw	CGAACTCTGCAGTATGACAAACGATACCACCTCGG	HVO_0686	*con. hyp. prot.*
		rev	GAGACGAGTTCCATCTCACTCACCTCCGTCGGCCGCG		
HVO_2555	*rps17*	fw	CGAACTCTGCAGTATGGCGATAGGACTTGACGTTC	HVO_2554	*rpl14*
		rev	CAGAGACGAGTTCCATCACTCGTCACCTCCCATAATCTCGACGACGAC		
b2488	*hyfH*	fw	CGATCCATGGTTGTGGGCGCAAGCGAGCGTC	b2489	*hyfI*
		rev	CGATCTCGAGGCTGACATGTTGTGTAAGCACTGG		
b1381	*ybdH*	fw	CGATCCATGGTTACGCTTTGGCGATAATCTCC	b1382	*ynbE*
		rev	GCATCTCGAGTGACGTCAACGCAGCCAG		
b0775	*bioB*	fw	CCAGCCCATGGACTGCCGTGCTGGCAGGGGATAAC	b0756	*bioF*
		rev	CCAGCCTCGAGCGCCGCGTTGATTTTCTCCTGCC		
b2264	*menD*	fw	CCAGCCCATGGTGGCGCACGCCAACCACCAC	b2263	*menH*
		rev	CCAGCCTCGAGTCCGTGTTTTGCCTGCGCGTG		
b1746	*astD*	fw	CCAGCCCATGTGGTATGCCGCAGATTACTGCGCAT	b1745	*astB*
		rev	CCAGCCTCGAGCCCGTCGAAATTGACTTCCCAGG		

Supplementary Table 4. Oligonucleotides for mutagenesis of intragenic Shine-Dalgarno sequences

HVO-Nummer	Gen upstream		Sequence (primer 5'-3‘ for mutagenesis)	HVO-Nummer	Gen downstream
HVO_0147	*ureB*	fw	GACGACGAACACGGCACCGCTAGTAGCGCGAGCGAATGACGAAGGAC	HVO_0148	*ureC*
		rev	GTCCTTCGTCATTCGCTCGCGCTACTAGCGGTGCCGTGTTCGTCGTC		
HVO_0357	*aa-bin. Prot.*	fw	CACGTCGTCGAACCGCTCGCTAGTAGCGCCCAATGAGCCTCAGACTCG	HVO_0358	*hom1*
		rev	CGAGTCTGAGGCTCATTGGGCGCTACTAGCGAGCGGTTCGACGACGTG		
HVO_2543	*rpl30*	fw	GAACAGATCGACGAACTCCTTAGTAGCGTGCGATGACGTCCAAGAAGC	HVO_2542	*rpl15*
		rev	GCTTCTTGGACGTCATCGCACGCTACTAAGGAGTTCGTCGATCTGTTC		
HVO_2551	*rpl5*	fw	CGAGTCCACGTTCGACGTTAGTAGCGAAGAATGAGCGATAGCGAAAC	HVO_2550	*rps15*
		rev	CGCTATCGCTCATTCTTCGCTACTAACGTCGAACGTGGACTCGATG		
HVO_1210	*flgA1*	fw	ACAACGACCCCATTGCGCTCTAGTAGCGTTCAAATGTTCAACAACATC	HVO_1211	*flgA2*
		rev	TTGTTGAACATTTGAACGCTACTAGAGCGCAATGGGGTCGTTGTTGTC		
HVO_2431	*glnP*	fw	GCTCGGATTGGCTCCTCAGCCGCCGATGACGCTC	HVO_2430	*glnQ*
		rev	ACGAGCGTCATCGGCGGCTGAGGAGCCAATCCGAGCG		
HVO_1594	*cna*	fw	AGCGTCATGCGGCTGAGGAGAGTTTGGCGGTGAAGAAG	HVO_1595	*con. hyp. prot.*
		rev	CACCGCCAAACTCTCCTCAGCCGCATGACGCTCGTCTC		
HVO_0685	*hyp. prot.*	fw	GTTCCATCTCACCTGAGGAGGTCGGCCGCGGCGGTGTC	HVO_0686	*con. hyp. prot.*
		rev	GCCGCGGCCGACCTCCTCAGGTGAGATGGAACTCGTC		
HVO_2555	*rps17*	fw	GTTCCATCACTCGCTGAGGAGCATAATCTCGACGACGAC	HVO_2554	*rpl14*
		rev	CGTCGAGATTATGCTCCTCAGCGAGTGATGGAACTCGTC		
b2488	*hyfH*	fw	GATGTACTGCTGGTGGGCAGAACCAACCTATGAGTCCAGTG	b2489	*hyfI*
		rev	CACTGGACTCATAGGTTGGCTGCCCACCAGCAGTACATC		
b1381	*ybdH*	fw	GCAAGGAAAAGAGTGTGCACCACTGCACAATGAAAATTTTACTG	b1382	*ynbE*
		rev	CGTTCCTTTTCTCACACGTGGTGACGTGTTACTTTTAAAATGAC		
b0775	*bioB*	fw	CGACGAATATTACACATTATTGTTGTTATGAGCTGGCAGG	b0756	*bioF*
		rev	CCTGCCAGCTCATAACAACAATAATGTGTAATATTCGTCG		
b2264	*menD*	fw	GCAACTTCTGGCGCAGGGCTGCTAGATTATGATCCTGCACG	b2263	*menH*
		rev	CGTGCAGGATCATAATCTAGCAGCCCTGCGCCAGAAGTTGC		
b1746	*astD*	fw	GGCTGGATTTTTCCGACACCACCTTGCGATGAACGCCTGG	b1745	*astB*
		rev	CCGACCTAAAAAGGCTGTGGTGGAACGCTACTTGCGGACC		

Supplementary Table 5. Oligonucleotides for amplification of dig-dUTP labeled probes for northern blot analysis of the reporter genes.

Reporter gene		Sequence (primer 5'-3‘ for probe amplification)
dhfr	fw	ATGACGCTCGTCTCTGTCGCCGCGCTC
	rev	AGGTCGTCGCGCATCGACTC
glpD	fw	CGCGGTTTATCCGTGCTGATGCTGGAGG
	rev	GCCGGTATCGATATCTTCCGCTTCCACAATCCAC
gusA	fw	GGGTGGACGATATCACCGTGGTGACG
	rev	CAATCACCACGATGCCATGTTCATCTGC

Supplementary Figure 1. Fractions of leading genes in 720 genomes of 24 groups of prokaryotes. Fractions of leading genes (monocistronic genes or first genes in operons). Mean values and standard deviations are shown.

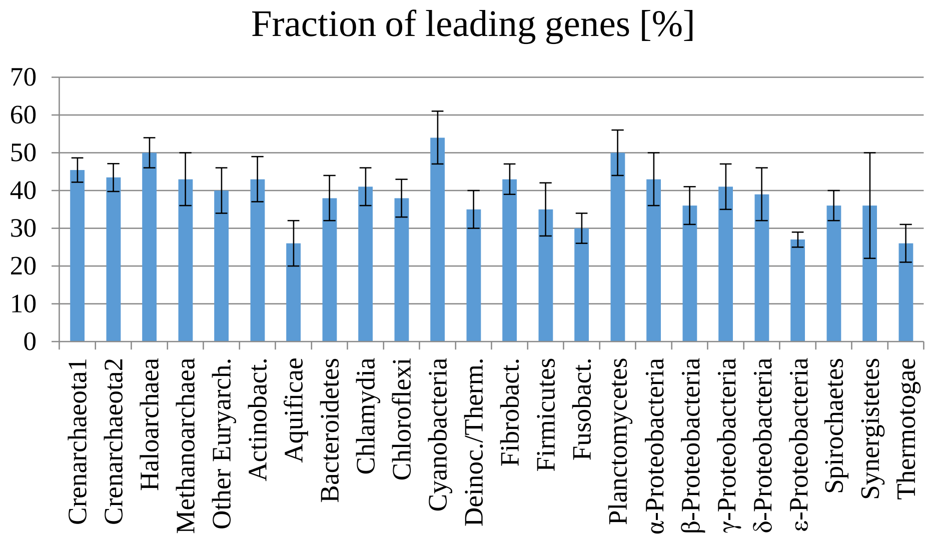

Supplementary Figure 2. (legend see next page)

A

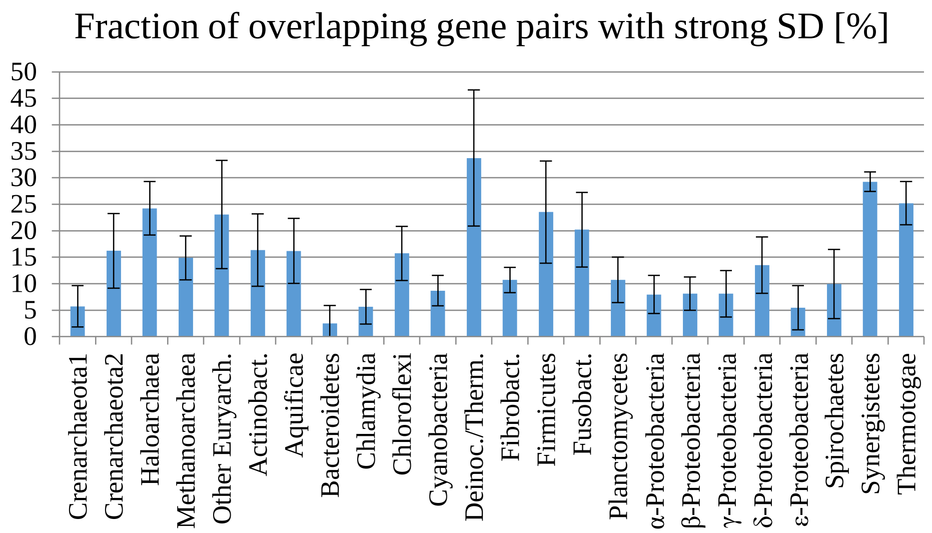

B

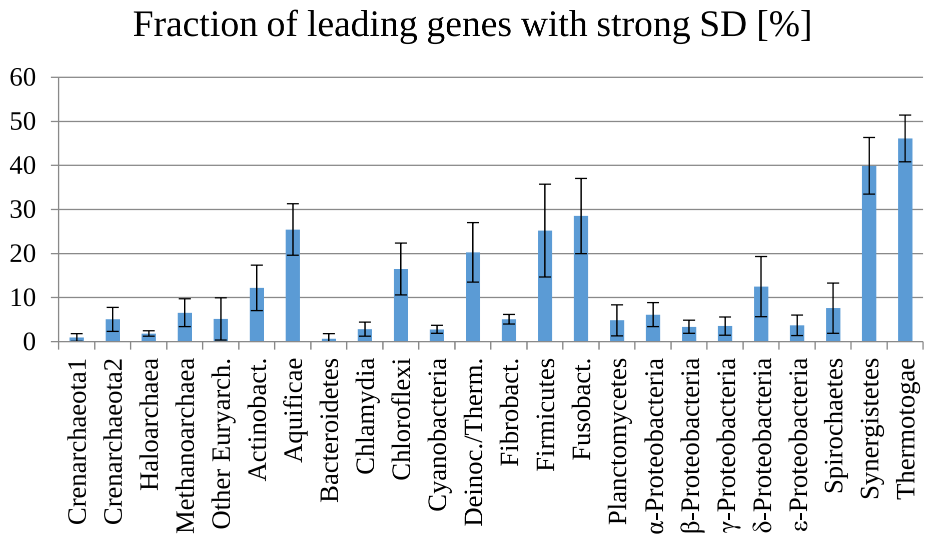

C

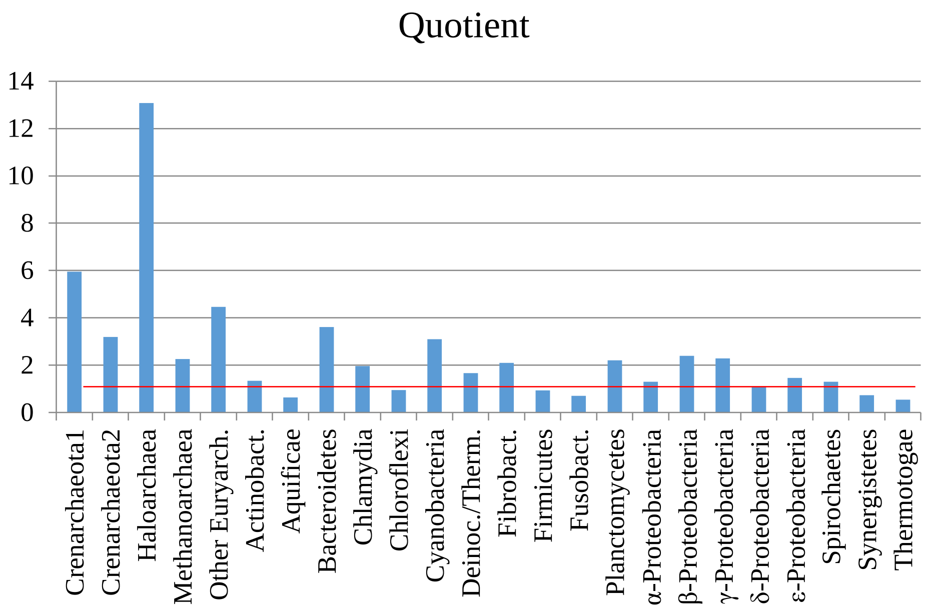

Supplementary Figure 2. Fractions of genes preceded by strong SD motifs with interaction energies of less than -8.4 kcal/mol.

A. Fractions of overlapping gene pairs with strong SD motifs in the 3'-region of the upstream gene.

B. Fractions of leading genes that are preceded by a strong SD motif. Average values and standard deviations are shown.

C. Quotients of the values shown in A and B. Values greater than one indicate that the strong SD motif is more important at overlapping gene pairs, values smaller than one indicate that the strong SD motif is more important at leading genes. Red horizontal line highlights a quotient value of one.

Supplementary Figure 3. A. Reporter enzyme activities and **B.** transcript levels used to calculate the translational efficiencies shown in Figures 4B and 4D.

A

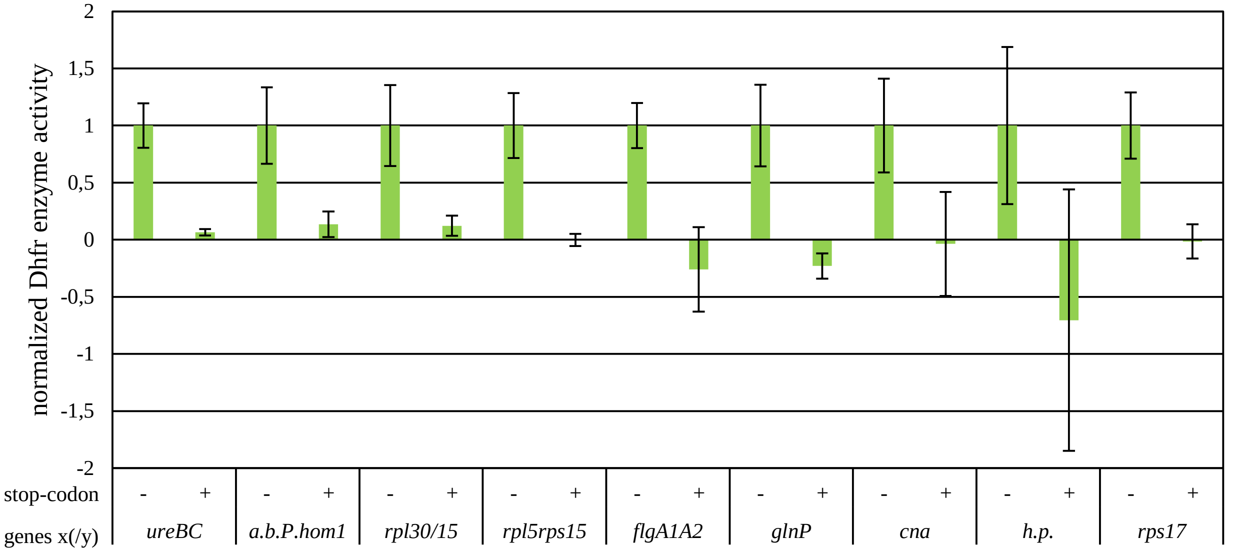

B

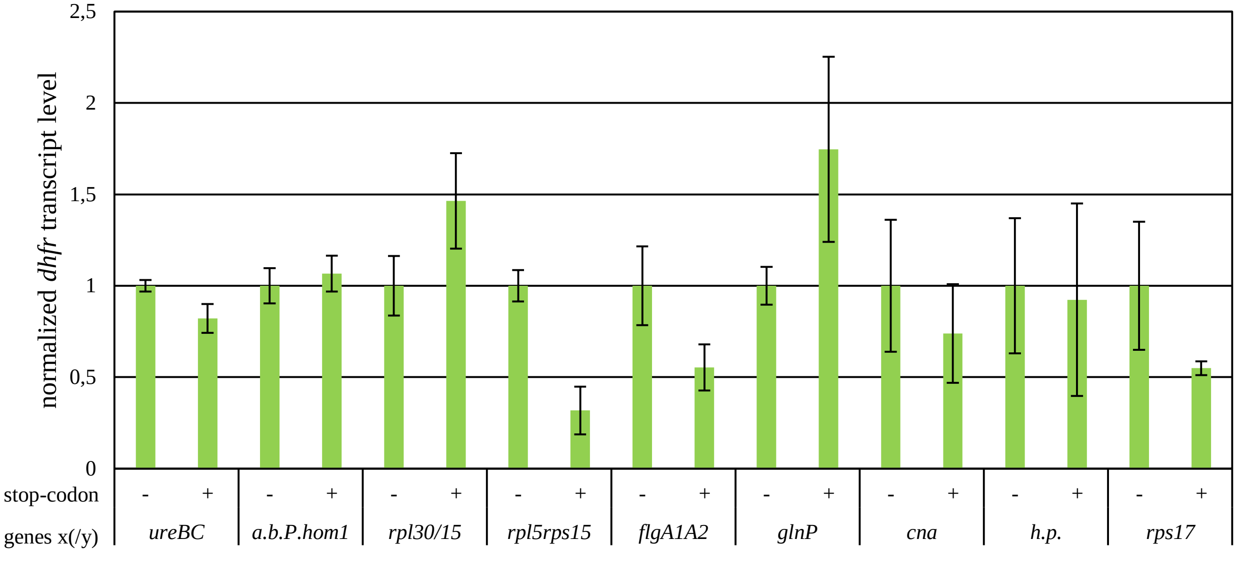

Supplementary Figure 4. Reporter enzyme activities (**A. C.)** and transcript levels (**B. D.**) used to calculate the translational efficiencies shown in Figures 4F and 4G.

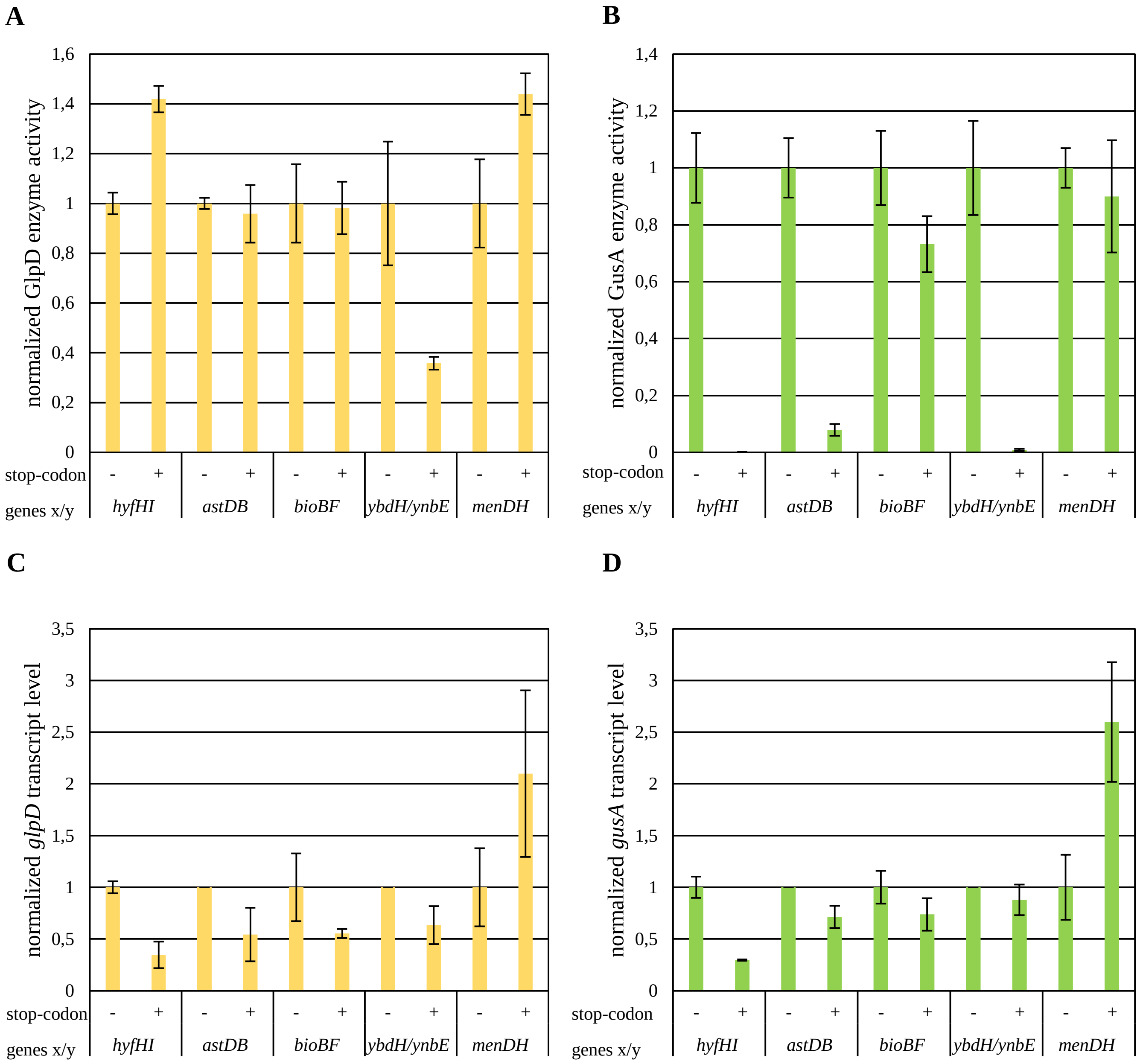

Supplementary Figure 5. A. Reporter enzyme activities and **B.** transcript levels used to calculate the translational efficiencies shown in Figures 5B and 5D.

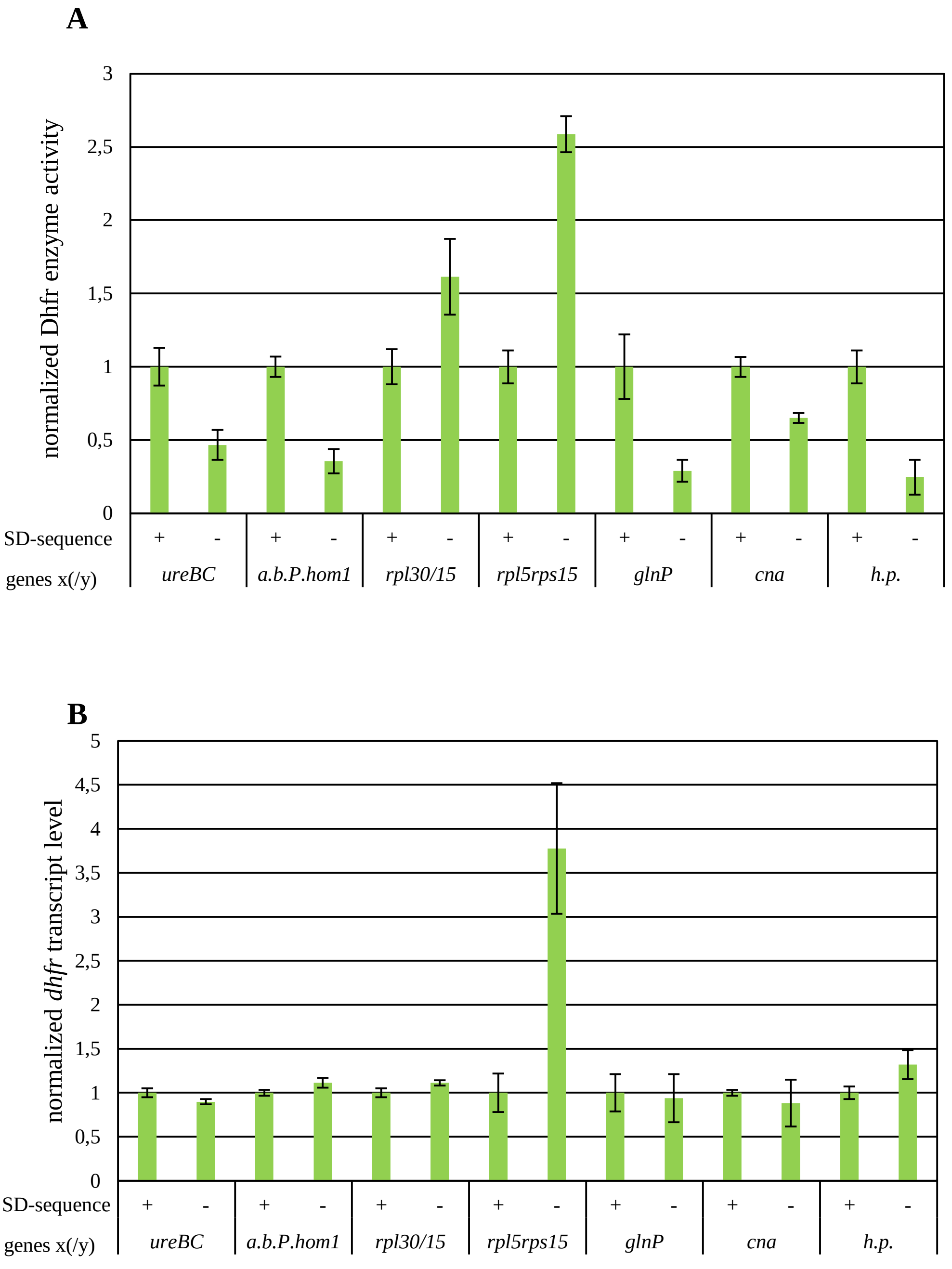

Supplementary Figure 6. Reporter enzyme activities (**A. C.**) and transcript levels (**B. D.**) used to calculate the translational efficiencies shown in Figures 5F and 5G.

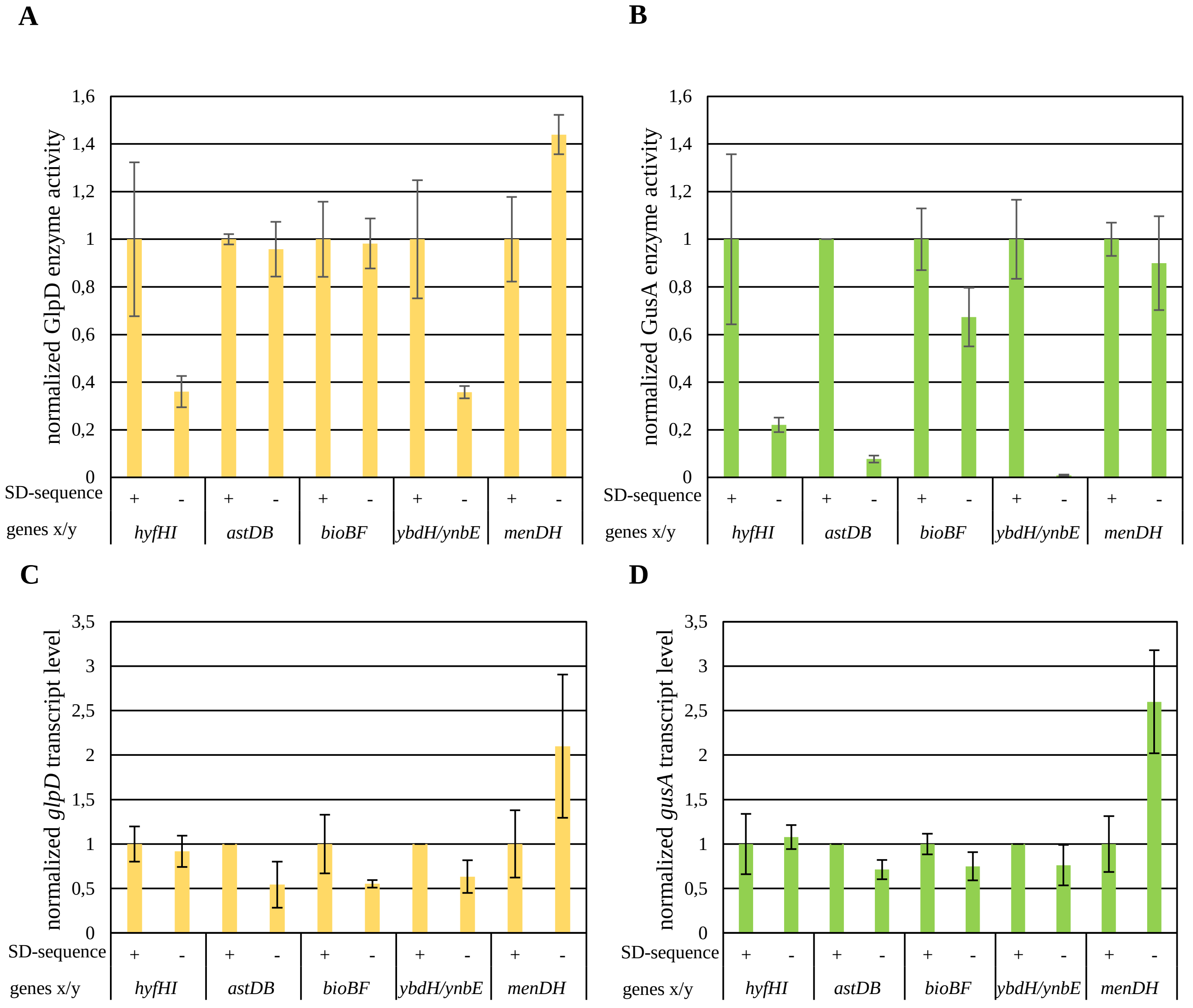

Supplementary Figure 7. *In silico* predicted structures of the cloned regions of the five overlapping gene pairs. The overlaps are boxed. The gene names are indicated.

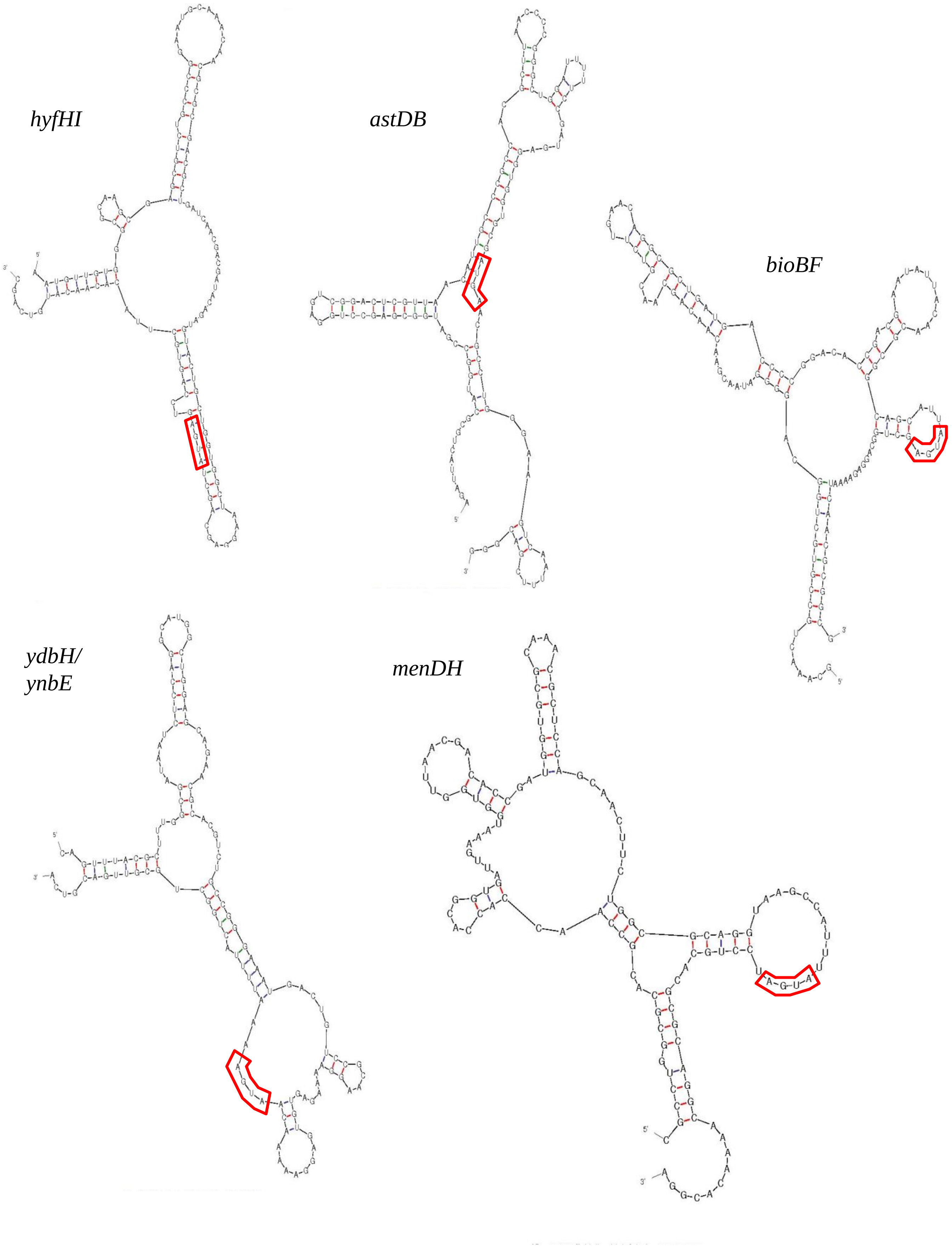

Supplementary Figure 8. Figure legend see next page.

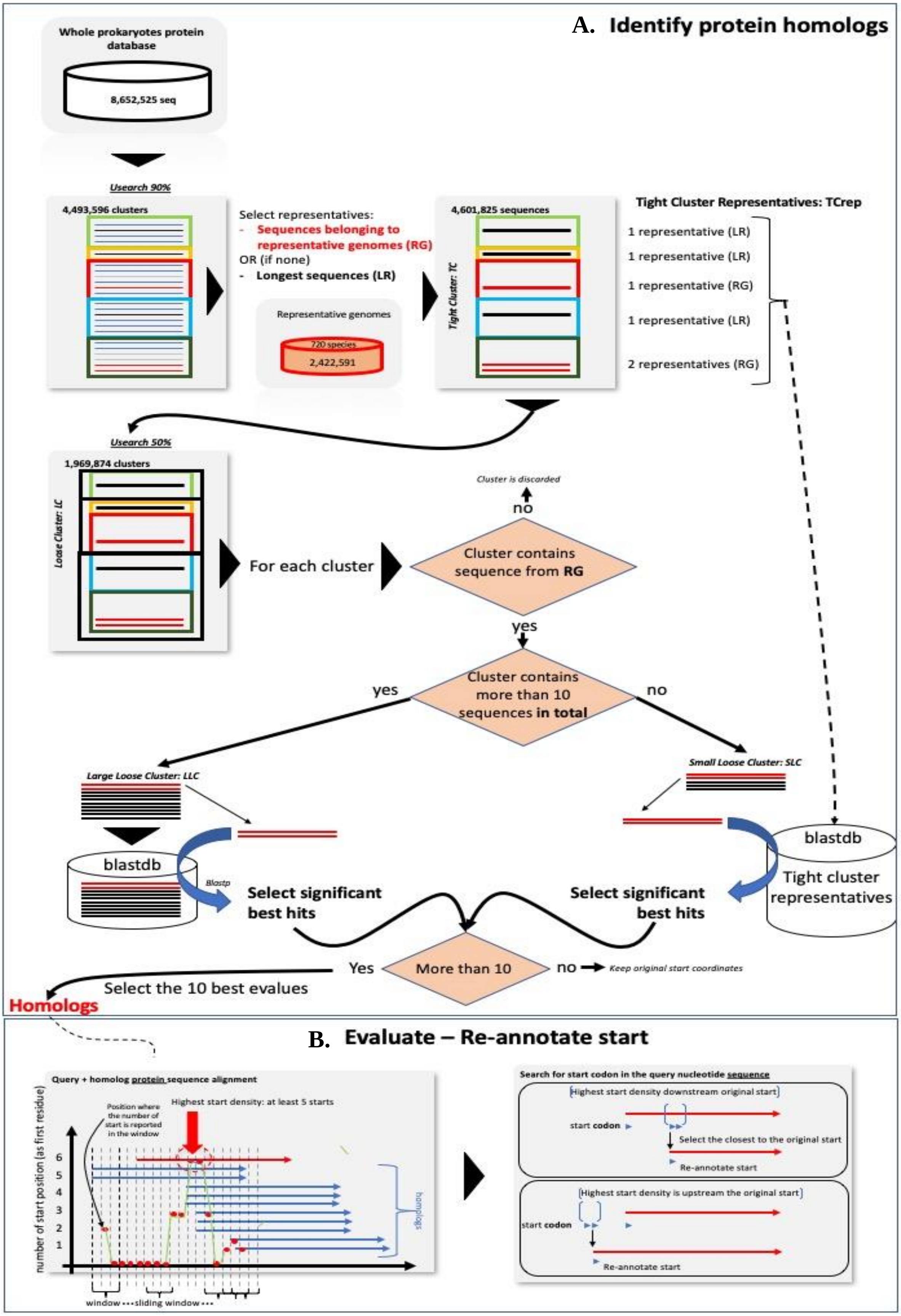

Supplementary Figure 8.

A. Identify protein homologs. Proteins are shown as red lines for protein sequences from the set of representative genomes and black lines for other prokaryotic proteins.

Tight clusters (TC) are embedded into bordered colored rectangles. Tight Cluster representatives (TCrep) can be either sequences from the representative genomes (RG) or the longest sequence of the Tight cluster (TC) when RG is not represented in the respective cluster. Loose clusters (LC) are indicated by bordered back rectangles containing one or several TC (colored rectangles).

The criteria employed to select protein clusters for further analysis are specified in diamonds.

Curved blue arrows indicated blastp procedures. Horizontal cylinders indicate protein sequence databases or blast databases (blastdb).

B. Evaluate – Re-annotate start. To the left, density of potential protein starts (the first residue of the respective protein) in each 3 position window of the protein alignments between the query (red arrow) and the homologous proteins (blue arrows).

The red dots indicated the number of protein starts throughout the alignment and within a specific window. The thick vertical arrow indicates the position in the alignment with the highest protein start density among the homologous proteins. Right, start reannotation strategies.

For each panel, top vertical arrow represents the original query nucleotide sequence, and the bottom arrow is the corrected start in the query ucleotide sequence. Brackets show the position with the highest protein start density mapped on the nucleotide sequence of the query;

blue triangles represent the start codon positions of the query nucleotide sequence in frame with the original, annotated start codon position.
