# Supplementary material for: Translational coupling via termination-reinitiation in archaea and bacteria
Source: Nat Commun. 2019 Sep 5;10:4006. doi: 10.1038/s41467-019-11999-9 (PMC6728339; doi:10.1038/s41467-019-11999-9)
Supplement: Supplementary file 3 — Description of Additional Supplementary Files [file 41467_2019_11999_MOESM3_ESM.pdf]

Description of Additional Supplementary Files

File Name: Supplementary Data 1
Description: Supplementary Data 1 summarizes the results of the bioinformatics analyses of 720 genomes of 24 groups of archaea and bacteria. The Table contains the fractions of overlapping gene pairs as well as the fractions of leading genes and of downstream genes in overlapping gene pairs that are preceded by a (strong) Shine Dalgarno motif.
